# Recurrent Abdominal Wall Abscess as an Atypical Presentation of Peritoneal Tuberculosis: A Case Report

**DOI:** 10.7759/cureus.85694

**Published:** 2025-06-10

**Authors:** Elizabeth Alcala, Pedro Hugo Guerrero Morales, José Manuel García Romero, Alejandro Morales Rubio, Santiago Andrés Rojas Guanoluisa

**Affiliations:** 1 Surgery, Hospital General de Queretaro, Querétaro, MEX; 2 Surgery, Hospital General de Querétaro, Querétaro, MEX

**Keywords:** abdominal abscess, extrapulmonary tuberculosis, general surgery, peritoneal tuberculosis (tb), tuberculosis

## Abstract

Peritoneal tuberculosis (TB) is an uncommon yet important form of extrapulmonary TB, often presenting a diagnostic challenge due to its nonspecific symptoms and diverse clinical manifestations. We report the case of a 38-year-old woman with type 2 diabetes mellitus, hypertension, chronic kidney disease, and a history of peritoneal dialysis, who presented with recurrent abdominal pain, fever, night sweats, and seropurulent discharge following prior abscess drainage. Despite empirical antibiotic therapy, her symptoms persisted. Imaging revealed intra-abdominal fluid collections and pneumoperitoneum, raising suspicion of intestinal perforation. Surgical exploration revealed a frozen abdomen with multiple cold abscesses, dense fibrous adhesions, and tubo-ovarian involvement. Histopathological examination confirmed peritoneal TB, showing caseous necrosis and Langhans giant cells. This case underscores the diagnostic complexity of tuberculous peritonitis, particularly in patients with a history of peritoneal dialysis and risk factors associated with endemic exposure to TB. Due to its overlap with intra-abdominal malignancies and other chronic infections, a high index of suspicion, histopathological confirmation - often via laparoscopy - and early initiation of anti-tuberculous therapy are critical for effective management and improved outcomes.

## Introduction

Tuberculosis (TB) remains a major global health concern, ranking among the top 10 causes of death worldwide and representing the leading cause of death among people living with HIV. The disease burden is substantial, with approximately 10 million new TB cases and 1.3 million deaths reported in 2018. In Mexico, approximately 25,000 cases of TB were reported in 2022, with extrapulmonary forms accounting for around 20-25% of cases, reflecting a persistent diagnostic and therapeutic challenge in endemic regions [1].

Abdominal TB is a rare form of extrapulmonary TB and can affect the gastrointestinal tract, peritoneum, lymph nodes, and solid organs. It may also coexist with conditions such as hepatic cirrhosis, carcinomatosis, sarcoma, or peritoneal dialysis [2,3].

Among the abdominal manifestations of TB, tuberculous peritonitis is a rare but notable entity, accounting for about 2% of all TB cases and 4-10% of extrapulmonary cases. It presents as a chronic, diffuse inflammatory condition caused by *Mycobacterium tuberculosis*, with nonspecific and often insidious symptoms, including abdominal pain, distension, fever, weight loss, and night sweats [2,3]. Definitive diagnosis requires microbiological or histopathological confirmation, with laparoscopy and peritoneal biopsy considered the gold standard due to their ability to provide both direct visualization and tissue sampling [3]. The subtle onset and indolent progression can lead to delayed medical evaluation and diagnosis, complicating clinical management.

Definitive diagnosis requires microbiological or histopathological confirmation, with laparoscopy and peritoneal biopsy considered the gold standard, as they allow direct visualization and tissue sampling [2,3]. Given the overlap with other intra-abdominal diseases, especially malignancies, peritoneal TB should remain a key differential in endemic areas and in patients with relevant risk factors.

In this context, we report the case of a 38-year-old woman with a history of chronic kidney disease and peritoneal dialysis, who developed an atypical presentation of peritoneal TB manifesting as a recurrent abdominal wall abscess.

## Case presentation

A 38-year-old female with a history of type 2 diabetes mellitus (managed with insulin), hypertension (treated with amlodipine), and chronic kidney disease on initial peritoneal dialysis (PD) presented with recurrent episodes of PD-associated peritonitis. Due to repeated infections, the PD catheter was removed in October 2023.

In December 2023, the patient underwent abdominal wall abscess drainage and received empirical antibiotics. However, despite incision and drainage, seropurulent discharge persisted from the midline site where the abscess had previously been drained. A private physician performed an office-based drainage procedure and prescribed doxycycline (100 mg every 12 hours) based on culture results.

With ongoing symptoms and recurrent abscess formation, the patient was admitted to our hospital in March 2024 with generalized abdominal pain of 10 days’ duration, associated with fever, nausea, vomiting, night sweats, and malaise.

A complete blood count revealed increased white blood cells, elevated lymphocyte count, and marked neutrophilia, suggestive of a systemic inflammatory or infectious process. Biochemical tests showed a marked elevation in serum creatinine (from a known baseline of 2.1 mg/dL to 7.0 mg/dL), fulfilling criteria for acute kidney injury in a patient with pre-existing chronic kidney disease. This rise was associated with oliguria and metabolic acidosis, supporting the clinical diagnosis. C-reactive protein levels were elevated, consistent with systemic inflammation. In addition, CA-125 levels were elevated, raising suspicion for peritoneal involvement (Table 1).

**Table 1:** Laboratory results

Laboratory test	Patient value	Reference range
White blood cell count (WBC)	15.27 × 10⁹/L	4.5–11.0 × 10⁹/L
Lymphocyte count	7.5 × 10⁹/L	1.0–4.8 × 10⁹/L
Neutrophil percentage	81.50%	40–60%
Serum creatinine	7.0 mg/dL	0.5–1.1 mg/dL (females)
C-reactive protein (CRP)	20 mg/L	<10 mg/L
CA-125	65 U/mL	0–35 U/mL

**Figure 1:**
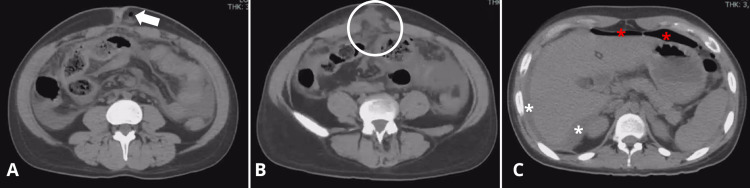
Non-contrast axial computed tomography of the abdomen showing an abdominal wall defect A: Axial CT image demonstrates a fluid collection and the presence of gas within the subcutaneous tissue at the level of the umbilical scar (white arrow). Additionally, free fluid is observed in the interloop spaces and along the left paracolic gutter. B: The image reveals a tract establishing communication between the intra-abdominal free fluid and the subcutaneous tissue of the abdominal wall (white circle), suggestive of a fistulous connection or abscess rupture. C: A water-density image shows perihepatic free fluid (white asterisks), and a hypodense area in the prehepatic region is suggestive of free intraperitoneal air (red asterisks), indicating possible bowel perforation.

Abdominal CT imaging demonstrated intra-abdominal fluid collections, perihepatic free fluid extending to the abdominal wall, and pneumoperitoneum, suggesting possible intestinal perforation (Figure 1).

Emergency exploratory laparotomy revealed a partially frozen abdomen (Zühlke III-IV fibrous adhesions), multiple cold abscesses in the pelvic cavity, adhesions among bowel loops, suspected peritoneal cold abscesses, and severe involvement of the right fallopian tube and ovary. No intestinal perforations or necrotic areas were found. Abscesses were drained, and a right salpingo-oophorectomy was performed.

Histopathology confirmed the presence of caseous necrosis, Langhans giant cells, foreign body-type giant cells, and both typical and atypical mycobacteria. The patient recovered postoperatively and was referred for follow-up by the epidemiology and infectious diseases departments.

## Discussion

Peritoneal TB, a manifestation of abdominal TB caused by *M. tuberculosis*, remains a diagnostic challenge due to its nonspecific presentation and limitations in conventional testing. It is the sixth most common site of extrapulmonary TB and accounts for approximately 50% of abdominal TB cases [1]. 

Underlying systemic diseases can compromise immune function, elevating the risk of *M. tuberculosis *infection and dissemination. Documented risk factors, including alcoholic liver disease, HIV infection, malignancy, anti-TNF therapy, and patients with end-stage renal disease undergoing continuous ambulatory peritoneal dialysis, promote cellular immune dysfunction, heightening susceptibility to TB [2].

Only 15-20% of abdominal TB cases show concurrent active pulmonary disease. Peritoneal TB most frequently arises from reactivation of a latent infection, while bacilli can reach the peritoneum via hematogenous spread from pulmonary foci to mesenteric lymph nodes, lymphatic rupture, ingestion of contaminated sputum or food, biliary excretion, or direct extension from adjacent organs or lymph nodes [2,4].

TB peritonitis typically follows an indolent clinical course, with progressive abdominal pain, distention secondary to ascites, and systemic manifestations such as weight loss, fever, and night sweats. The gradual symptom progression causes patients to often experience symptoms for several months before seeking medical attention, frequently leading to severe complications, including abscess formation, intestinal obstruction or perforation, and disseminated disease [5]. 

Peritoneal involvement can be broadly classified into three main types based on the predominant pathological features, although these patterns often overlap, resulting in a dynamic and evolving clinical presentation. The wet (ascitic) type is characterized by large volumes of free or loculated ascites with high protein content, typically producing an enhanced peritoneal appearance on imaging. By contrast, the dry (plastic) type is marked by a fibrotic response, with nodular peritoneal thickening and the formation of adhesions. The fixed fibrotic type primarily affects the omentum and mesentery, often extending to the bowel serosa. Imaging in these cases commonly reveals matted bowel loops, omental caking or masses, and loculated ascites, findings suggestive of chronic fibrosis [5]. This patient’s findings were consistent with the fibro-adhesive type.

Ascitic fluid analysis is key to diagnosis and often reveals straw-colored, lymphocyte-predominant exudate with elevated protein (>25 g/L) and a low serum-ascites albumin gradient (SAAG <1.1 g/dL). Adenosine deaminase (ADA) levels >30 IU/L show >90% sensitivity and specificity for peritoneal TB. Interferon-gamma (>3.2 U/mL) in ascitic fluid offers similar diagnostic performance. However, AFB staining and culture have limited sensitivity (3% and <20%, respectively) [6,7].

Imaging assessment with ultrasound and CT commonly demonstrates free, loculated, or localized ascites (36-67%), lymphadenopathy (14-47%), and peritoneal thickening (23-32%) [2,8]. CT imaging often reveals high-attenuation ascites, peritoneal thickening, omental caking, and mesenteric lymphadenopathy. These findings, combined with elevated CA-125 levels, can mimic ovarian malignancies or carcinomatosis, leading to potential misdiagnosis and unnecessary surgeries [3]. 

In cases of wet-type peritoneal TB, characterized by prominent, viscous ascites, an initial diagnostic approach may involve ultrasonography-guided paracentesis for fluid analysis. The preferred gold standard for diagnosis is Mycobacterial culture from a peritoneal biopsy, laparoscopy with histopathology, and culture of peritoneal biopsies has a sensitivity approaching almost 100% for detecting peritoneal TB [2,7].

Laparoscopy enables direct visualization of peritoneal and omental nodules, adhesions, and ascites, along with biopsy confirmation. Typical findings include thickened peritoneum, yellow-white nodules, and calcified lymph nodes, recommended in patients with unexplained ascites and high clinical suspicion of peritoneal TB [7].

Early diagnosis and prompt initiation of treatment are crucial in tuberculous peritonitis, as delays in treatment initiation have been consistently linked to increased mortality. The standard treatment follows extrapulmonary TB guidelines, primarily involving systemic anti-TB drug regimens, supplemented by nutritional support and localized abdominal therapy [9,10]. In cases of significant exudative ascites, therapeutic paracentesis may be performed, followed by intraperitoneal administration of isoniazid and streptomycin. Surgical intervention is reserved for complications such as complete intestinal obstruction, bleeding, intestinal fistula, or perforation. In such cases, abscess drainage combined with anti-TB therapy is essential for achieving a cure and minimizing the risk of disease recurrence. This combined approach ensures optimal outcomes while addressing both infectious and structural complications of peritoneal TB [2,7].

## Conclusions

This case illustrates the diagnostic complexity of peritoneal TB, especially in patients with prior peritoneal dialysis and comorbid conditions such as diabetes and chronic kidney disease. The insidious and nonspecific clinical presentation of peritoneal TB often overlaps with that of various intra-abdominal pathologies, such as malignancies, pelvic inflammatory disease, or peritoneal carcinomatosis. While not all intra-abdominal conditions present nonspecifically, the clinical similarity in early stages can lead to diagnostic confusion and delays. Moreover, imaging and laboratory markers such as elevated CA-125 levels may mimic findings seen in malignancy, further complicating the clinical picture. Laparoscopy with histopathological confirmation remains essential for diagnosis. High clinical suspicion, especially in TB-endemic regions, combined with early surgical intervention and initiation of anti-tuberculous therapy, is key to improving outcomes and preventing complications. Clinicians should maintain a high index of suspicion for tuberculous peritonitis in at-risk populations presenting with recurrent or atypical abdominal symptoms.
